# Reply to Nachamkin, “Diversity of *Campylobacter* species in a rhesus macaque breeding colony”

**DOI:** 10.1128/msphere.00041-25

**Published:** 2025-03-25

**Authors:** Rebecca L. Bacon, Carolyn L. Hodo, Sara D. Lawhon

**Affiliations:** 1 Department of Pathology, School of Medicine, and Division of Laboratory Animal Resources, Duke University 189411 , Durham, North Carolina, USA; 2 Michale E. Keeling Center for Comparative Medicine and Research, The University of Texas MD Anderson Cancer Center 151235 , Bastrop, Texas, USA; 3 Department of Veterinary Pathobiology, School of Veterinary Medicine & Biomedical Sciences, Texas A&M University 199063 , College Station, Texas, USA; University of Michigan-Ann Arbor 1259 , Ann Arbor, Michigan, USA

**Keywords:** *Campylobacter jejuni*, *Campylobacter coli*, *Campylobacter*, Rhesus macaque, macaque, chronic enterocolitis, diarrhea

## REPLY

We appreciate Dr. Irving Nachamkin’s perspective on the complicated topic of diarrheal illness in rhesus macaques. We agree that additional approaches to more fully understand the contribution of other curved and spiral bacteria species to health and illness in rhesus macaques are needed. In addition to the many potential pathogens mentioned by Dr. Nachamkin, bacteria in this category may have roles in the normal gastrointestinal tract of rhesus macaques, which could be equally important in understanding disease processes in this species. For example, *Helicobacter macacae* was originally implicated as a potential pathogen in rhesus macaques but has since been shown to represent a normal commensal organism whose absence is indicative of dysbiosis (1, 2).

This study was subject to some limitations which required narrowing the field of investigation to *Campylobacter jejuni* and *C. coli*. Funding availability was a consideration, but, additionally, the processing of nonhuman primate (NHP) fecal samples requires special precautions due to potential serious zoonotic pathogens such as Herpesvirus simiae, also called Herpes B or B virus (https://www.cdc.gov/herpes-b-virus/about/index.html). Some veterinary laboratories, including the Clinical Microbiology Laboratory at the Texas A&M University Veterinary Medical Teaching Hospital, do not allow acceptance of NHP samples. For this reason, we were restricted to culture using the existing protocols of the clinical microbiology laboratory at the NHP facility from which samples were collected. Some of these limitations were addressed in the manuscript, including likely selection of thermophilic species and laboratory standard operating procedures limiting colony selection to one isolate per sample. With these considerations, *C. jejuni* and *C. coli* were chosen as the bacteria of interest for this study given their association with post-infectious irritable bowel syndrome (PI-IBS) in humans and, as such, their potential role in the development of chronic enterocolitis in macaques. While *Helicobacter pylori* is a known causative agent of gastritis and occasionally colitis in rhesus macaques, chronic enterocolitis/idiopathic colitis has not been shown to be associated with any type of specific gastritis (3), and pathologists at the Keeling Center where this colony of macaques is housed have not observed a correlation between the presence of gastritis and the chronic enterocolitis syndrome (C. L. Hodo and personal communications). The role of *H. pylori* in the development of PI-IBS in humans is also unclear, so it was not selected for further investigation in this study (4).

We agree wholeheartedly with Dr. Nachamkin’s recommendation for expanded molecular approaches in the survey of gastrointestinal health of nonhuman primate populations. Preliminary results of DNA extracted from 55 fecal swab specimens from this cohort subjected to PCR using *Campylobacter* sp.-specific 16S primers demonstrated 100% positivity (average Ct value = 21.3). However, 37/55 (67.3%) of those samples were PCR-negative for *C. jejuni* and *C. coli*. All but one of those 37 samples were also culture-negative, suggesting the presence of *Campylobacter* sp. not detected by our other methods. Determining the identity of these species is an active area of investigation, as initial cloning and Sanger sequencing efforts have been unfruitful. Microbiome analyses from this NHP colony, though not this specific study population, are also in progress and are a priority area for future research efforts.

Ultimately, our goal was to focus on two known enteric pathogens with clearly defined roles in the development of PI-IBS to determine any similarities in the rhesus colony and development of chronic diarrhea, and we acknowledge the many opportunities for further research that exist to more completely address this question. We are excited about the interest in enteric bacterial populations of concern in rhesus macaques that this paper has generated and look forward to future studies investigating as yet unidentified bacterial species in this and other rhesus macaque colonies.
